# Potential Protective Effect of Oleanolic Acid on the Components of Metabolic Syndrome: A Systematic Review

**DOI:** 10.3390/jcm8091294

**Published:** 2019-08-23

**Authors:** Ángel Fernández-Aparicio, Jacqueline Schmidt-RioValle, Javier S. Perona, María Correa-Rodríguez, Jose M. Castellano, Emilio González-Jiménez

**Affiliations:** 1 Department of Nursing, Faculty of Health Sciences, University of Granada, Av. Ilustración, 60, 18016 Granada, Spain; 2 Instituto de la Grasa, Spanish National Research Council (CSIC), Campus University Pablo de Olavide, 41013 Seville, Spain

**Keywords:** triterpenes, metabolic syndrome, insulin resistance, hypertension, inflammation, obesity

## Abstract

The high prevalence of obesity is a serious public health problem in today’s world. Both obesity and insulin resistance favor the development of metabolic syndrome (MetS), which is associated with a number of pathologies, especially type 2 diabetes mellitus, and cardiovascular diseases. This serious problem highlights the need to search for new natural compounds to be employed in therapeutic and preventive strategies, such as oleanolic acid (OA). This research aimed to systematically review the effects of OA on the main components of MetS as well as oxidative stress in clinical trials and experimental animal studies. Databases searched included PubMed, Medline, Web of Science, Scopus, EMBASE, Cochrane, and CINAHL from 2013 to 2019. Thus, both animal studies (*n* = 23) and human clinical trials (*n* = 1) were included in our review to assess the effects of OA formulations on parameters concerning insulin resistance and the MetS components. The methodological quality assessment was performed through using the SYRCLE’s Risk of Bias for animal studies and the Jadad scale. According to the studies in our review, OA improves blood pressure levels, hypertriglyceridemia, hyperglycemia, oxidative stress, and insulin resistance. Although there is scientific evidence that OA has beneficial effects in the prevention and treatment of MetS and insulin resistance, more experimental studies and randomized clinical trials are needed to guarantee its effectiveness.

## 1. Introduction

The increasing prevalence of overweight and obesity entails a serious global public health problem. It has been estimated that 39% of the world population over the age of 18 years is overweight, and 13% is obese, according to World Health Organization (WHO) data (2016) [1]. Furthermore, 18% of children and adolescents (5–19 years old) are either overweight or obese [1,2]. This situation is alarming because obesity and insulin resistance are two of the most important factors leading to metabolic syndrome (MetS) [3]. MetS consists of a set of cardiometabolic anomalies that lead to the development of type 2 diabetes mellitus (T2DM) and of cardiovascular disease (CVD) [4,5]. Nevertheless, MetS is also associated with other clinical conditions such as oxidative stress, hypertension, dyslipidemia, hepatic steatosis, non-alcoholic fatty liver disease, and impaired glucose tolerance, among others [6].

According to the WHO [5], insulin resistance is the main pathophysiological factor underlying MetS. It is characterized by a diminished tissue response to the cell activity of insulin [6], which implies a reduction in glucose uptake in adipocytes and muscle cells, an increase in hepatic glucose production, and altered lipid metabolism in the liver and adipose tissue [7]. Various studies highlight the relationship of insulin resistance to obesity and inflammation. Obesity-associated hyperplasia and hypertrophy of adipose tissue cause an increase in proinflammatory cytokines [8], such as tumor necrosis factor alpha (TNF-α) and interleukin-6 (IL-6) [9]. These proinflammatory cytokines, which have a negative effect on insulin signaling, are regulated by nuclear transcription factor kappa B (NF-kB), one of the pathways that activates oxidative stress [10].

The various definitions of MetS proposed by organizations such as the WHO, the European Group for the Study of Insulin Resistance (EGIR), and the National Cholesterol Education Program—Adult Treatment Panel III all underline that central MetS components are abdominal obesity, insulin resistance, hypertension, and dyslipidemia [3,5]. In this same line, in 2006, the International Diabetes Federation (IDF) defined MetS as “central obesity plus any two of the following four factors: raised triglycerides, reduced HDL cholesterol, raised blood pressure, and raised fasting plasma glucose.” Accordingly, the treatment and prevention of MetS should not only be envisaged as a whole, but each MetS component should also be considered individually.

The high prevalence of obesity and MetS because of sedentary and generally unhealthy life styles [1,2] makes the application of pharmacological interventions necessary, which have the tendency to be expensive for the health care systems. In addition, some of these drugs have several side effects that adversely affect the quality of life. In this sense, therapeutic properties of bioactive compounds are increasingly being studied [11,12]. It has been reported that oleanolic acid (OA), a naturally occurring pentacyclic triterpenoid, found at a high content in the leaves and fruit of the olive tree, among other plants [13], has various interesting pharmacological properties for the prevention and treatment of MetS and insulin resistance, such as anti-inflammatory, anti-oxidant, hypolipidemic, antidiabetic, anti-atherosclerotic [10,13,14] and antihypertensive [14,15] effects. The mechanisms of action that support these properties have been studied by different authors. OA is a selective Takeda G-protein-coupled receptor 5 (TGR5) agonist, whose activation have beneficial effects on glucose homeostasis, proinflammatory cytokines and body weight [16]. Furthermore, other authors refer that OA could suppresses the NF-kB and activate the nuclear factor erythroid 2–related factor 2 (Nrf2) signaling pathways, both having important roles on the inflammatory status in insulin resistance [10,17].

Consequently, as a bioactive compound, OA has potential to be considered for the development of new and alternative therapeutic strategies for insulin resistance and MetS. Therefore, this study was aimed to systematically review the effects of OA formulations on the components of MetS and on proinflammatory cytokines and antioxidant enzymes as oxidative stress biomarkers in human subjects and animal models. 

## 2. Materials and Methods 

### 2.1. Databases and Search Strategy

In June 2019, a systematic review was performed in accordance with the Preferred Reporting Items for Systematic Reviews and Meta-Analyses (PRISMA) 2009 guidelines [18]. The objective was to systematically identify the clinical trials and experimental studies in animals that have evaluated the effects of OA on insulin resistance and the various MetS components to date. For this purpose, we conducted a bibliographic search with a time filter from January 2013 until June 2019 in the following five electronic databases: Medline, Web of Science, Scopus, EMBASE, Cochrane and CINAHL. PubMed search engine was also consulted.

The search terms were based on the following descriptors in the Medical Subject Headings (MeSH): oleanolic acid, metabolic syndrome, insulin resistance, obesity, hypertension, and inflammation. The search strategy used in all of the databases was Oleanolic acid AND (“metabolic syndrome” OR “insulin resistance” OR obesity OR hypertension OR inflammation).

### 2.2. Selection of Papers. Eligibility Criteria

The studies were selected in two phases. In the first phase, all titles and abstracts were read and analyzed in order to select the most potentially relevant studies, based on the following inclusion criteria: (1) oleanolic acid administration, (2) focus on insulin resistance and/or MetS components, (3) clinical trials in humans and experimental studies on animals, (4) articles published in English, and (5) access to the full text. In doubtful cases, the complete text of the article was analyzed.

In the second phase, we analyzed the full text of the articles selected in the previous phase in order to determine their eligibility. The articles excluded in this phase had one of the following criteria: (1) secondary studies, (2) no use of OA or use of an OA derivative, (3) combined administration of OA with another bioactive compounds, or (4) research based on the molecular study of OA and its biological activity. This selection process was performed by two independent reviewers (A.F-A. and J.S-R.), though a third reviewer (J.S.P.) was also consulted in doubtful cases.

### 2.3. Data Extraction

After selecting the studies for the qualitative synthesis of this systematic review, the next step was to extract the data. Data extracted both from the clinical trial and the animal studies were the following: authors and publication year, subjects, sample size, type of intervention, duration of the intervention, dosage used, and outcomes obtained (hypertension, lipid profile, hyperglycemia, insulin resistance, and inflammatory and oxidative stress biomarkers). Data extraction was performed by two independent reviewers (E.G-J. and M.C-R.), though a third reviewer (J.M.C.) was consulted in cases of doubt. Each of the results measured was described in a narrative form. A meta-analysis of the animal studies included was ruled out because of the heterogeneous nature of the studies, especially in reference to their design and the animal species used in these studies.

### 2.4. Risk of Bias and Methodological Quality Assessment

To reduce inter-examiner bias, two independent reviewers (A.F-A. and J.S-R.) performed the methodological quality assessment and analyzed the risk of bias of all the studies included in this review. When there was any doubt, a third reviewer (J.S.P.) was consulted. The methodological quality assessment of the animal intervention studies was performed by using the SYRCLE’s Risk of Bias (RoB) tool [19], whereas the Jadad scale was used to evaluate the methodological quality of the clinical trial [20].

SYRCLE’s RoB tool was elaborated by the Systematic Review Centre for Laboratory Animal Experimentation (SYRCLE) [19] for assessing the methodological quality of experimental studies of animals and is based on the Cochrane Collaboration RoB tool. SYRCLE’s RoB tool contains 10 items, five of which (i.e., items 1, 3, 8, 9, and 10) coincide with those of the Cochrane RoB tool because they are applicable to animal experiments. The remaining items were adapted to the characteristics of animal experimentation studies. These items assess six types of bias: selection bias, performance bias, detection bias, attrition bias, reporting bias and another bias. In SYRCLE’s RoB tool each of the 10 items is rated to a “yes” (low risk of bias), a “no” (high risk of bias), or “unclear” (insufficient information to evaluate risk of bias). 

The Jadad scale [20] is a five-item tool used for reporting risk of bias of clinical trials and each of its items assess randomization, method of randomization, double-blinding, method of blinding and reporting of withdrawals, respectively. The Jadad score for ranges from 0 to 5, 0 being the lowest level of quality and 5 the highest. 

## 3. Results

### 3.1. Study Selection

**Figure 1:**
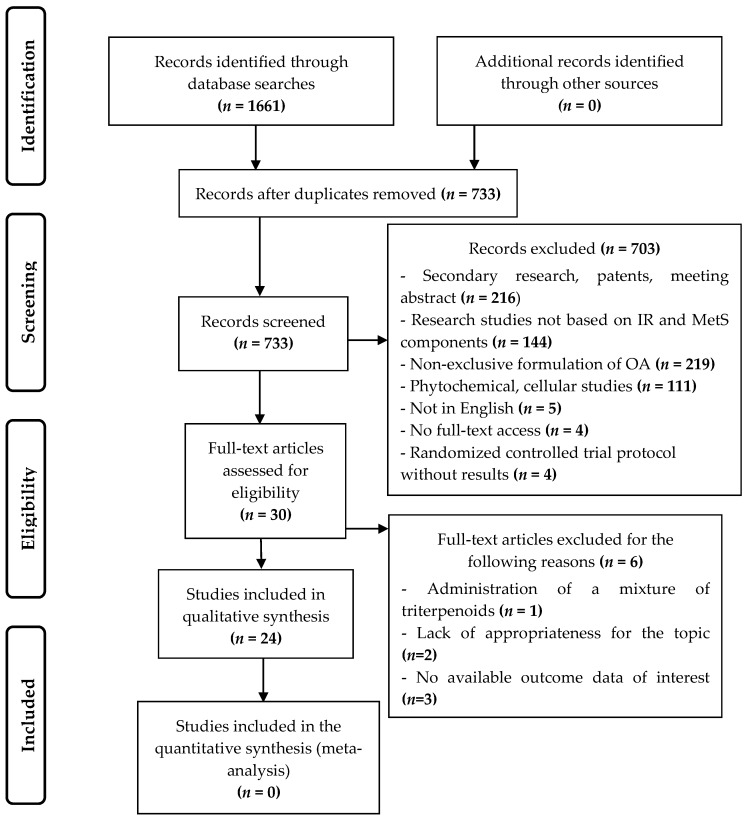
Flow diagram according to the PRISMA Checklist. Selection of studies. OA: oleanolic acid; IR: Insulin resistance; MetS: Metabolic syndrome.

The search strategy, implemented in the various databases and the PubMed search engine, with a time filter from January 2013 until June 2019, produced the following 1661 results: 523 from the Web of Science, 382 from Scopus, 271 from Medline, 286 from EMBASE, 91 from PubMed, 101 from CINAHL, and 7 from Cochrane. After the duplicated publications were eliminated, the titles and abstracts of 733 articles were analyzed to ascertain whether they fulfilled the inclusion criteria. Full texts of 30 articles were then read in order to assess their suitability for the study. Finally, 24 of these articles were included in the systematic review without carrying out an inverse search of the literature. Figure 1 shows the flow diagram of the selection and exclusion process of the research studies according to the PRISMA system [18].

In the first phase of selection, four studies were excluded because of having not full-text access [21,22,23,24]. It is also noteworthy that two randomized double-blind controlled trials that studied the effects of virgin olive oils enriched with bioactive compounds, such as phenolic compounds and different triterpenes on MetS and oxidative stress were found in the first phase of selection [25,26]. However, they were excluded because the aim of this systematic review is to evaluate the effects of OA alone, without interactions with another bioactive compounds.

### 3.2. Characteristics of the Animal Studies Selected

Table 1 summarizes the characteristics of the animal studies selected. In addition, this table includes the results of these studies on the effect of OA on insulin resistance and MetS components. Of the animal studies selected, the majority (*n* = 20) used rodents, particularly rats and mice. The experimental animals in the remaining studies were quails (*n* = 1), rabbits (*n* = 1) and a mixed study of rabbits and mice (*n* = 1). In the studies that used rats and mice, the largest sample size was *n* = 122 and the smallest sample size was *n* = 18. The sample size in the study of quails was *n* = 120; in the study of rabbits, *n* = 24; and in the mixed study, there were 32 rabbits and 56 mice. The minimum OA dosage administered was 5 mg/kg and the maximum OA dosage was 250 mg/kg. The maximum administration time period was 20 weeks, and the minimum time period was one week. 

### 3.3. Characteristics of the Clinical Trial Selected 

Table 2 summarizes the characteristics of the clinical trial selected.

### 3.4. Risk of Bias and Methodological Quality Assessment

According to the authors of the SYRCLE’s RoB tool, the risk of bias assessment should be presented as a table or a figure that gives either the summary results of the assessment or the results of all individual studies. They do not recommend calculating a summary score for each individual study when using this tool since this inevitably involves assigning “weights” to specific domains in the tool, and it is difficult to justify the weights assigned [19]. Accordingly, Table 3 presents the results obtained with SYRCLE’s RoB tool for each study. In addition, the Jadad score is presented in Table 4.

### 3.5. OA Effects on Insulin Resistance and MetS Components in Animal Studies 

#### 3.5.1. Hypertension

Three studies were performed in hypertensive animal models [27,28,29]. Ahn et al. [27] reported that the application of OA over a period of three months produced a significant decrease in systolic blood pressure (SBP) in hypertensive rats in comparison to non-OA-treated hypertensive rats. In this same line, the administration of OA by Bachhav et al. [28] to Nω-nitro-L-arginine methyl ester (L-NAME)-induced hypertensive rats for four weeks significantly reduced SBP and the mean arterial blood pressure (MAP). It also significantly increased urine volume and urine sodium excreted, as well as non-significantly increased serum nitrate/nitrite (NOx) levels in comparison to the group of rats that was only given L-NAME. Similarly, in a study conducted by Madlala et al. [29], the administration of various doses of OA (30, 60, and 120 mg/kg) significantly reduced MAP from the third week until the end of the intervention in OA-treated rats in comparison to the control group. Moreover, the results obtained in this study indicated a significant increase in urine sodium excretion, but not in the volume of urine excreted. Furthermore, in the study conducted by Gamede et al. [30], a reduction of the MAP was observed in prediabetic rats. In summary, OA reduced SBP and MAP and increased urinary excretion of sodium. 

.

#### 3.5.2. Lipid Profile and Obesity

In a study performed by Chen et al. [31], the administration of OA over a four-week period produced a significant decrease in serum levels of triglycerides (TG), total cholesterol (TC), LDL, and HDL in diabetic mice in comparison to non-OA-treated diabetic mice. 

One mixed-animal study and two trials that used non-rats/mice animal models assessed the effects of OA on high-fat diet (HFD)-induced atherosclerosis [32,33,34]. In the study conducted by Jiang et al. [32], it was reported that all OA-treated quails fed along with a HFD for 10 weeks experienced a significant reduction in TG, TC, and LDL, as well as a significant increase in HDL and nitric oxide (NO) serum levels. The experimental study conducted by Luo et al. [33] for 12 weeks was performed on rabbits, LDL receptor knockout (LDLR−/−) mice, and C57BL/6J mice. Only the experimental groups of these animal models received OA in the last five weeks of the study. The OA-treated rabbits showed a significant reduction in TG, TC, and LDL levels, with a slight increase in HDL levels. OA-treated (LDLR−/−) mice experienced a significant decrease in TG and LDL levels, as well as a significant increase in HDL compared to the HFD control group. Finally, the OA-treated C57BL/6J mice not only showed a significant reduction in TC and LDL levels but also no changes in their HDL and TG levels in comparison to non-OA-treated HFD mice. At the end of their study, Pan et al. [34] reported that there was a significant reduction in TG, TC, LDL, and HDL levels in the OA-treated rabbits. 

The administration of OA to diabetic mice by Wang et al. [35] showed a significant decrease of TG, TC, LDL, and free fatty acids (FFA) serum levels and a significant increase of HDL levels in comparison to non-OA-treated diabetic mice. Similarly, in the study conducted by Gamede et al. [30], OA significantly reduced body weight, TG, and LDL plasma levels, and also led to a significant increase of HDL levels in prediabetic rats in comparison to non-OA-treated prediabetic rats.

Molepo et al. [12] administered OA to neonatal rats fed with a HF diet and observed a decrease of saturated FFA, and an increase of mono/polyunsaturated FFA. OA administration by Nakajima et al. [41] significantly reduced the plasma levels of octanoylated ghrelin levels and the body weight gain in comparison to non-OA-fed rats.

Therefore, according to the results reported, OA improved lipid profile, as evidenced by the decrease in serum levels of TG, TC, and LDL. However, regarding serum HDL levels, some studies reported an increase, while others reported a decrease.

#### 3.5.3. Hyperglycemia and Insulin Resistance

Wang et al. [35] showed that, after administrating OA to diabetic mice, body weight, fasting blood glucose (FBG), and fasting serum insulin (FSI) decreased significantly. The results of the glucose area under the curve (AUC), obtained from the intraperitoneal glucose tolerance test (IPGTT) and intraperitoneal insulin tolerance test (IPITT) (both performed after the intervention), were significantly lower in diabetic OA-treated mice than in those that had not received OA. Similarly, in the study performed by Li et al. [36] in fructose-induced insulin-resistant rats treated with OA, an oral glucose tolerance test (OGTT) was performed on the eighth day of the intervention, and it was observed that the AUC for FFA was significantly lower, whereas AUC for glucose experienced a slight decrease. Moreover, they also found a significant reduction of the homeostatic model assessment of insulin resistance (HOMA-IR) and the adipose tissue insulin resistance (Adipo-IR) scores in comparison to non OA–treated insulin-resistant rats. 

Lee et al. [37] administered OA for 20 weeks to type 2 diabetic rats and observed a significant increase in the homeostasis model assessment of β-cell function (HOMA-β) and insulinemia in comparison to non-OA-treated type 2 diabetic rats. Furthermore, the OA-treated rats were found to have a slightly lower glucose AUC after the IPGTT and the intravenous insulin tolerance test (IVITT). Similarly, a study performed on diabetic mice by Wang et al. [38] showed that OA administration significantly reduced FBG, HOMA-IR, and serum HDL levels as well as non-significantly reduced FSI in comparison to the control group. Moreover, there was a significant improvement in glucose AUC. 

Two similar studies were conducted by Gamede et al. [30,39] in high-fat high-carbohydrate (HFHC) diet-induced prediabetic rats. In both studies, rats were administered OA after prediabetes induction. During OA administration, the rats continued to receive either an HFHC diet or a normal diet. In the first study [39], both OA-treated groups experienced a significant reduction in body weight, in glycemia in the OGTT test and in the HOMA2-IR and HbA1c indexes as well as a reduction in the hepatic and muscle glycogen concentration in comparison to the non-OA-treated prediabetic rats. In the other study [30] a reduction of FBG was observed in comparison to non-OA-treated prediabetic rats. 

Djeziri et al. [40] showed that after administrating OA for 16 weeks to HFD-induced obese mice, a signification reduction in glycemia levels at 15, 30, 60, and 120 min after the administration of glucose was observed in the IPGTT test in comparison to non-OA-treated HFD-induced obese mice. In addition, from six week until the end of the intervention, the increase in body weight of the OA-treated mice was significantly lower than the HFD control group.

Su et al. [42] administered OA to polychlorinated biphenyls (PCBs)-induced metabolic disfunction mice and observed a significant decrease of FBG, FSI, HOMA-IR, adipocyte size, and serum levels of TG, FFA, and TC. It was also observed a significant decrease of glycemia in the IPGTT and IPITT tests. In the study performed by Wang et al. [43] in high fat and fructose (HFF) diet-fed rats, OA administration reduced significantly body weight, FBG, FSI, and serum levels of NO and TG, as well as increased the insulin sensitivity index (ISI).

OA administration by An et al. [44] to diabetic rats with a carotid artery injury showed a significant reduction of levels of FBG and a significant increase of serum NO level in comparison to non-OA-treated diabetic rats with the same injury.

Two studies performed by Nyakudya et al. [46,47] assessed the protective long-term effects of neonatal intake of OA in rats. These animals were administered OA, a high-fructose (HF) solution, or OA combined with the HF solution during their second week of life. From postnatal day 56 until the end of the experiment, half of each group received either distilled water or a fructose-rich solution. At the end of the first experiment [46], a significant increase in glucose in the OGTT test was observed in both administration periods in comparison to all of the OA-treated female rats. Moreover, both HF solution-fed male and female rats had a significant higher HOMA-IR index than their respective OA-fed male and female rats in both periods. In the another study [47], a significant increase of hepatic lipid content in male rats, and a significant raise in terminal body mass in female rats fed with a HF solution, was observed in both periods in comparison to their respective OA-treated rats. 

In summary, the reported results showed that OA improved the AUC of glucose and insulin in the glucose tolerance tests, decreased the serum levels of FBG and FSI and reduced the HOMA-IR index.

#### 3.5.4. Inflammatory and oxidative stress biomarkers. Antioxidant enzymes

In the studies conducted by Pan et al. [34], Wang et al. [35] and An et al. [44], the administration of OA led to significant decreases in serum levels of IL-1β, IL-6, and TNFα. Similarly, a reduction of plasma levels of IL-6 and TNFα was observed by Gamede et al. [30]. In agreement with these results, a significant reduction in the gene expression of IL-1β and IL-6 in liver and adipose tissue, as well as of TNFα in adipose tissue of mice was observed by Djeziri et al. [40]. In addition, Matumba et al. [45] administered OA to neonatal rats fed with a high fructose (HF) diet, and they observed a decrease of IL-6 and TNFα plasma levels and a reduction of their gene expression at the end of the experiment. An increase of plasma level of adiponectin was also observed by these authors [45]. In summary, OA was able to decrease both the serum levels and gene expression of the proinflammatory cytokines IL-1β, IL-6, and TNFα.

A number of authors showed an increase of serum superoxide dismutase (SOD) activity [30,32,37,42,43]. In addition to SOD, Jiang et al. [32] and Gamede et al. [30] also reported an increase in glutathione peroxidase (GSH-Px) activity. Moreover, the administration of OA in the study conducted by Madlala et al. [29] decreased malonaldehyde (MDA) concentration in the heart, liver, and kidney and increased the activities of SOD and GSH-Px in the liver and kidney. In this same line, a reduction of heart MDA concentration was found in the study of Gamede et al. [30], as well as a decrease of serum MDA in the studies conducted by Jiang et al. [32] and Wang et al. [43]. Furthermore, Jiang et al. [32], Su et al. [42], and Wang et al. [43] also showed an increase of serum catalase (CAT) activity. Moreover, Nyakudya et al. [48] administered OA to neonatal rats fed with a HF diet and observed a decrease of MDA concentration, as well as an increase of CAT activity by the end of the study. Accordingly, OA increased the activity of antioxidant enzymes and decreased MDA levels.

### 3.6. Hypolipidemic Effects of OA in Human Patients

In a study performed by Luo et al. [49], OA administration for four weeks to hyperlipidemic patients elicited a decrease of TC, TG, LDL, glucose, and FSI serum levels, as well as an increase of leptin serum levels. A slight decrease of HbA1c (%) and a slight increase of HDL was also observed. 

## 4. Discussion

The aim of this systematic review was to investigate the effects of OA on parameters concerning on components of Mets, including central obesity, lipid profile, blood pressure, hyperglycemia, as wells as insulin resistance and/or oxidative stress biomarkers. The findings provided in this study derive mostly from experimental studies in animals, while only one non-randomized clinical trial in humans was included. The main findings of this study are (i) OA administration improves the hypertensive status, (ii) the disturbance of the lipid profile in hyperlipidemic and metabolic dysfunction situations is attenuated by OA, (iii) OA reduces the oxidative stress status, and (iv) the insulin resistance condition is improved by the action of OA. Taken together, these findings suggest that OA has potential to be a new or alternative therapeutic strategy to the insulin resistance and metabolic syndrome treatments.

In this review, some studies showed that OA improves hypertension, one of the Mets components. More specifically, in the studies of Ahn et al. [27] and Bachhav et al. [28], there was a significant reduction of SBP. Additionally, a significant decrease of MAP was reported by Bachhav et al. [28], Madlala et al. [29], and Gamede et al. [30]. This improvement in hypertension could be due to the reported hypotensive effect of OA, probably by the modulation of the renin-angiotensin-aldosterone system and the synthesis of atrial natriuretic peptide [27,50], since Bachhav et al. [28] and Madlala et al. [29] showed that OA increased the quantity of urine sodium excreted [27,50]. Another possible hypotensive mechanism of action of OA could be the increase of the production of nitric oxide (NO), a vasodilator factor that is diminished in endothelial disfunction induced by cardiovascular risk factors such as hypertension, obesity, diabetes, and dyslipidemia [15]. This is in agreement with the increase of serum NO levels produced by the action of OA reported by Bachhav et al. [28], Jiang et al. [32], and An et al. [44]. However, Wang et al. [43] reported the opposite, which might be explained by the fact that they studied rats [43] fed with a high fat and fructose diet. High fructose diets are closely linked to a higher oxidative stress status [51], which increases NO through enhancing inducible nitric oxide synthase (iNOS) expression [52,53].

With regard to the lipid profile, the significant reduction in TG and LDL serum levels [30,31,32,33,34,35], as well as the significant increase in HDL serum levels [30,32,33,35] observed in animal studies [30,32,33,35] suggest that OA could prevent oxidative stress-induced CVD, since low HDL levels and high LDL levels in obesity caused an overproduction of reactive oxygen species (ROS), especially in obesity-induced oxidative stress [54]. However, in the studies carried out by Chen et al. [31], Pan et al. [34], and Wang et al. [38], OA significantly decreased serum HDL levels. These differences found in HDL serum levels might be explained by the different animal models or clinical contexts studied, as well as the different OA dosage applied. Interestingly, the decrease in serum levels of TG and LDL in hyperlipidemic patients treated with OA reported by Luo et al. [49] is consistent with all the results obtained in animals. However, the slight increase of HDL serum levels in hyperlipidemic patients is only in agreement with the results from animal studies by Gamede et al. [30], Jiang et al. [32], Luo et al. [33], and Wang et al. [38].

Dysregulation of adipokines plays a main role in the association between insulin resistance and oxidative stress [55]. Matumba et al. [45] showed that OA enhanced adiponectin plasma concentrations, which could result on greater insulin sensitivity [56] due to its insulin-sensitizing properties through enhancing hepatic IRS-2 expression [57]. In the present review, we found that OA increased serum leptin levels in hyperlipidemic patients [49], an adipokine that favors the synthesis of proinflammatory cytokines [54]. This result of OA in leptin serum levels might be due to an impairing leptin signaling in hyperlipidemic conditions [58]. Although the mechanism by which OA reduces body weight is not yet clear, two of the studies that reported a decrease in body weight also observed a reduction of ghrelin levels (Gamede et al. [39] and Nakajima et al. [41]). Ghrelin has been shown to induce body weight gain through increasing food intake [59]. 

On the other hand, the reduction of plasma levels of FFA [12,35,42] as well as the lower Adipo-IR index [36] caused by the action of OA, might imply an increase of insulin secretion because chronic exposure to elevated levels of FFA leads to ROS overproduction [60] and thus to impaired insulin signaling and beta-cell failure [54,55]. In this line, the literature supports that OA increases insulin biosynthesis and secretion [10], which is in accordance with the increased insulin levels and HOMA-β index [37], as well as the reduced FSI levels [35,36,38,43]. In addition, Li et al. [33], Wang et al. [35], Su et al. [37], and Nyakudya et al. [40] reported an improvement in the HOMA-IR index, whereas Gamede et al. [41] reported an improvement of the HOMA-2 IR index. These findings might be explained by the availability of OA to modulate insulin signaling pathways such as glycogen synthase (GS)/glycogen phosphorylase (GP) signaling pathway [61,62] or the insulin receptor substrate 1 (IRS1)-glucose transporter 4 (GLUT4) pathway via NF-κB [17]. Interestingly, a synthetic-biology-inspired therapeutic strategy based on OA-triggered short human glucagon-like-peptide 1 (GLP-1) expression through TGR5 pathways has been successfully developed and applied in hepatogenous diabetic mice [63]. Nonetheless, according to the literature, OA improves beta-cell function through increasing insulin biosynthesis and secretion and also improves glucose tolerance [10]. This idea coincides with different studies included in our review that reported improvements of glucose tolerance both in glucose [35,36,38,39,40,42,46] and in insulin [35,42] tolerance tests, as well as reductions of FBG [30,35,38,42,43,44].

It is noteworthy that the decrease in both serum levels [30,34,35,44,45] and gene expressions [40,45] of proinflammatory cytokines reported in our review, probably due to the modulatory effect of OA on NF-kB [17], could result in an improvement of insulin secretion and beta-cell function because proinflammatory cytokines, whose levels are higher in obese individuals, have an important role in the development of insulin resistance [8,9,10]. Moreover, [17,30,34,35,40,44,45].

The ability of OA to alleviate oxidative stress and to improve pancreatic beta-cell function could also be related to the increase of SOD [29,30,32,37,42,43], GSH-Px [29,30,32], and CAT [32,42,43,48] activities, as well as the reduction of MDA [29,30,32,43,48]. Since OA might activate the transcription factor Nrf2, which increases the transcription of antioxidant enzymes (SOD, CAT, and GSH-Px), the aforementioned variations observed in the antioxidant enzymes might be explained by a possible activation of Nrf2 by OA [9,10].

Therefore, OA has potential effects on the components of MetS and insulin resistance. However, the results reported in our review have shown that OA has inconsistent effects on serum levels of HDL and NO, since they are increased in some studies and decreased in others. In this sense, these parameters should be further investigated, and the animal models and clinical situations studied should be standardized to better understand the effects of OA. Thus, a better understanding of the effects of OA may allow more randomized controlled trials to be carried out. 

This study has some strengths and limitations. One of its strengths is the use of the PRISMA 2009 checklist, one of the most prestigious sets of guidelines for the reporting of systematic reviews [18]. Another strength is the use of the SYRCLE’s Risk of Bias (RoB) tool and the Jadad scale for assessing the methodological quality of the animal studies and the clinical trial included, respectively. Moreover, the initial selection of studies, data extraction, and the evaluation of the studies finally included in our review were performed by two independent reviewers, who consulted a third reviewer when there was any doubt in order to reduce the risk of subjectivity [64,65]. Furthermore, a wide number of databases were consulted. This review has some limitations, such as that unpublished material sources were not consulted, which might have resulted in a selection bias [66]. Another limitation is the presence of only one clinical trial and its non-randomized design.

## 5. Conclusions

In summary, from the data from studies in experimental animals assessed in the present systematic review, we conclude that OA administration may improve hypertension, attenuate the disturbance of the lipid profile in metabolic dysfunction situations, reduce oxidative stress status, and improve insulin resistance. In reference to the non-randomized clinical trial assessed in the present work, we conclude that OA may improve the hyperlipidemic status, as well as glycemia in hyperlipidemic patients. These findings confirm the potential of the OA to be effectively used in the treatment of the MetS and insulin resistance. However, there is need for performing further animal studies that include all parameters involved in the development of insulin resistance and MetS in order to provide a more in-depth understanding of the OA effects on these metabolic disorders. More importantly, it is peremptory to conduct randomized clinical trials whose results will open the door to the possible use of OA in humans as a therapeutic alternative or as a complement to conventional therapy.

## Figures and Tables

**Table 1:** Characteristics and results of animal experimentation studies on the effect of oleanolic acid (OA) on insulin resistance and metabolic syndrome (MetS) components.

Author/Year	Subjects	Sample Size (n)	Intervention	Dosage	Duration	Results
Ahn YM et al. [27] (2017)	Hypertensive (HTA) and normotensive rats	31	Oleanolic acid (OA) by oral gavage	30 mg/kg/day	7 weeks (OA last 3 weeks)	↓ SBP (*p* < 0.001) in OA-treated HTA rats vs. HTA control
Bachhav SS et al. [28] (2015)	L-NAME, during the intervention, induced hypertensive rats	34	Oral administration of OA	60 mg/kg/day	4 weeks	↓ SBP (*p* < 0.001) and MAP (*p* < 0.05), ↑ urine excretion and urine sodium vs. L-NAME control group; ↓ non-significant (ns) of body weight and ↑ ns of serum NOx vs. L-NAME control group.
Madlala HP et al. [29] (2015)	Normotense, DSS and SHR rats	18	Oral administration of OA	30, 60 and 120 mg/kg twice every three days	9 weeks	↓ MAP (*p* < 0.05), ↑ urine sodium excretion in DSS and SHR rats; ↓MDA in al tissues, and ↑ SOD and GSH-Px activities in liver and kidney in DSS and SHR rats, only in OA60
Chen S et al. [31] (2017)	HFD-fed mice and diabetic db/db mice	20	Intraperitoneal injection of OA	20 mg/kg b.w/day	4 weeks	↓ TG, TC, LDL, HDL (*p* < 0.05) in OA-treated diabetic mice vs. non-OA-treated diabetic mice
Jiang Q et al. [32] (2015)	HFD-fed quails	120	OA via gavage	25, 50 and 100 mg/kg/day	10 weeks	↓ serum TG, TC, LDL and MDA, and ↑ HDL (4.05 ± 0.31 vs. 2.63 ± 0.52 mM, *p* < 0.05), NO (37.60 ± 9.15 vs. 29.49 ± 7.47 µM, *p* < 0.05), SOD, CAT, GSH and GSH-Px vs. HFD control group, especially with 100 mg/kg of OA.
Luo H et al. [33] (2017)	32 rabbits 32 C57BL/6J mice 24 LDLR−/− mice	88	OA administration to animals fed with atherogenic diet	10 (rabbits) and 25 (mice) mg/kg/day	12 weeks (last 5 weeks OA)	↓ TG, TC, LDL vs. non-OA-treated rabbits ↓ TG, LDL, ↑ HDL vs. non-OA-treated LDLR−/− mice ↓ TC, LDL vs. non-OA-treated C57BL/6J mice.
Pan Y et al. [34] (2018)	HFD-fed rabbits	24	OA via gavage	50 mg/kg/day	12 weeks (last 4 weeks OA)	↓ TG (*p* < 0.001), TC (*p* < 0.001), LDL (*p* < 0.05) and HDL (*p* < 0.01); ↓ serum levels of IL-1β, IL-6 (*p* < 0.001), and TNFα (*p* < 0.001) vs. HFD control group.
Molepo M et al. [12] (2018)	Pups rats.	96	OA via oral gavage	60 mg/kg/day	16 weeks (2nd week OA)	↓ saturated FFA, and ↑ mono/polyunsaturated FFA vs. control group
Wang X et al. [35] (2013)	Non-diabetic rats and diabetic mice	34	Intraperitoneal injection of OA	20 mg/kg/day	2 weeks	↓ FBG, and FSI; ↓ body weight (36.4 ± 2.3 vs. 41.7 ± 4.1 g); ↓ TG, TC, LDL, FFA, IL-1β, IL-6, and TNFα, and ↑ HDL both in serum and ↓ liver;↓ AUC of IPGTT and IPITT. All changes (*p* < 0.05) vs. non-OA-treated diabetic mice.
Li Y et al. [36] (2014)	Fructose induced insulin resistant rats	24	Oral administration of OA	5 and 25 mg/kg/day	10 weeks	↓ FSI, HOMA-IR and Adipo-IR vs. non-OA-treated insulin resistant rats; ↓ AUC of FFA and ↓ non-significant of glucose in the OGTT vs. non-insulin resistant rats. These changes (*p* < 0.05) only with OA 25 mg.
Lee ES et al. [37] (2016)	Non-diabetic and T2DM rats	-	OA via oral gavage	100 mg/kg/day	20 weeks	↓ Body weight vs. non-diabetic rats control group. ↑ Insulinemia, HOMA-β and serum SOD, and ↓ TG vs. non-OA-treated diabetic rats.
Wang X et al. [38] (2015)	Diabetic mice	24	Intragastric administration of OA	250 mg/kg/day	4 weeks	↓ FBG (*p* < 0.001), HOMA-IR (*p* < 0.05) and HDL (7.54 ± 0.82 vs. 9.02 ± 0.97 mM/l, *p* < 0.01), improved glucose AUC of OGTT and ↓ non-significant of FSI vs. control group
Gamede M et al. [39] (2018)	HFHC diet induced prediabetic rats	36	Oral administration of OA	80 mg/kg/3days	12 weeks	↓ Body weight (*p* < 0.05), glycemia in the OGTT (*p* < 0.05), HOMA2-IR (60.35 ± 2.05 vs. 128.26 ± 2.98, *p* < 0.05), HbA1c, ghrelin, hepatic and muscular glycogen concentration vs. non-OA-treated prediabetic rats.
Gamede M et al. [30] (2019)	HFHC diet induced prediabetic rats	36	Oral administration of OA	Not mentioned	12 weeks	↓ Body weight (516.75 ± 8.28 vs. 679.75 ± 78.52 g), FBG, MAP, and plasma levels of TG, LDL, IL-6 and TNF-α, ↑ plasma level of HDL (1.88 ± 0.02 vs. 0.85 ± 0.04 mM/l), SOD and GSH-Px, and ↓ heart MDA concentration vs. prediabetic control group. All changes *p* < 0.05
Djeziri FZ et al. [40] (2018)	HFD induced obese mice	18	Oral administration of OA	Not mentioned	16 weeks	↓ Glycemia in the IPGTT; and ↓ gene expression of IL-1β, IL-6, and TNFα vs. HFD control group
Nakajima K et al. [41] (2019)	STD, HFD or HGD-fed mice	18	OA by oral gavage	20 and 40 mg/kg/day	1 week	↓ plasma octanoylated ghrelin levels and body weight gain in STD-fed rats vs. non-OA-treated STD-fed rats
Su S et al. [42] (2018)	PCBs-induced metabolic disfunction in mice	40	Oral administration of OA	50 mg/kg/3days	10 weeks	↓ FBG (132 ± 14 vs. 191 ± 16 mg/dl), HOMA-IR (1.02 ± 0.17 vs. 1.79 ± 0.35) and serum levels of TG, FFA, cholesterol and FSI (1.35 ± 0.41 vs. 2.8 ± 0.56 ng/dl); ↓ Glucose level in IPGTT and IPITT. All changes (*p* < 0.05) vs. non-OA-treated PCBs-induced mice
Wang S et al. [43] (2018)	HFF diet-fed rats	36	OA and Nano-OA by gavage	25mg/kg/day	12 weeks (last 6 weeks OA)	↓ BW, FBG and serum NO level, ↑ serum CAT activity in OA and nano-OA groups. ↓ serum levels of FSI, TG and MDA, ↑ ISI and serum SOD activity in nano-OA group. All changes (*p* < 0.05) vs. non-treated insulin resistant rats.
An Q et al. [44] (2017)	Streptozotocin-induced diabetic rats.	18	Oleanolic acid	100 mg/kg/day	12 weeks (last 6 weeks OA)	↓ FBG, serum levels of IL-1β (*p* < 0,001), IL-6 (*p* < 0.05), and TNFα (*p* < 0.01); ↑ serum NO level (*p* < 0.01) vs. non-OA-treated diabetic rats.
Matumba MG et al. [45] (2019)	Pups rats	40	Neonatal OA administration by orogastric gavage	60 mg/kg/day	16 weeks (2nd week OA)	↑ Adiponectin (1,5 fold, *p* < 0.01); ↓ IL-6 (*p* < 0.01) and TNFα plasma concentration; and ↓ gene expression of IL-6 (*p* < 0.0001) and TNFα *p* < 0.0001) vs. non-OA-treated HF-fed rats
Nyakudya TT et al. [46] (2018)	Pups rats. High fructose to half of the rats	112	Neonatal OA administration	60 mg/kg/day b.w.	16 weeks (2nd week OA)	↓ AUC in the OGTT, and of the HOMA-IR index in the rats treated with OA.
Nyakudya et al. [47] (2018)	Pups rats	112	Neonatal OA administration	60 mg/kg/day b.w.	16 weeks (2nd week OA)	↑ hepatic lipid content in male rats, and in terminal body mass in female rats fed with HF as neonates and as a adults vs. OA-treated rats.
Nyakudya et al. [48] (2019)	Pups rats in their second postnatal week	30	Neonatal OA administration by orogastric gavage	60 mg/kg/day b.w.	1 week	↑ level of GSH and CAT activity, ↓ MDA concentration in skeletal muscle tissue vs. HF-fed rats.

Notes: All results referenced are statistically significant unless otherwise noted; SBP, systolic blood pressure; MAP, mean arterial blood pressure; L-NAME, Nω-nitro-L-arginine methyl ester; NOx, serum nitrate/nitrite level; DSS, Dahl salt-sensitive; SHR, spontaneously hypertensive rats; MDA, malonaldehyde; SOD, superoxide dismutase; GSH-Px, glutathione peroxidase; HFD, high-fat diet; TG, triglycerides; TC, total cholesterol; LDL, low-density lipoprotein; HDL, high-density lipoprotein; NO, nitric oxide; CAT, catalase; GSH, total glutathione; FFA, free fatty acids; FBG, fasting blood glucose; FSI, fasting serum insulin; AUC, area under the curve; IPGTT, intraperitoneal glucose tolerance test; IPITT, intraperitoneal insulin tolerance test; T2DM: 2 type diabetes mellitus; OGTT, oral glucose tolerance test; HFHC, high-fat high-carbohydrate; HOMA-IR and HOMA2-IR: insulin resistance index; STD: standard diet; HGD, high-glucose diet; PCBs, polychlorinated biphenyls; HFF, High fat and fructose; BW, body weight; ISI, insulin sensitivity index; HF, high fructose. ↑ means reduction/decrease, etc of the results; and ↓ means an increase.

**Table 2:** Characteristics and results of the clinical trial on the effect of OA.

Author/Year	Subjects	Sample Size (n)	Intervention	Dosage	Duration	Results
Luo HQ et al. [49] (2018)	Hyperlipidemic patients	15	Oleanolic acid	Not mentioned	4 weeks	↓ TC, TG, LDL, glucose and FSI;
						↑ HDL and Leptin; slight ↓ of HbA1c

Notes: TC, total cholesterol; TG, triglycerides; LDL, Low-density lipoprotein; HDL, High-density lipoprotein; FSI, fasting serum insulin; HbA1c, glycosylated hemoglobin A1c. ↑ means reduction/decrease, etc of the results; and ↓ means an increase.

**Table 3:** SYRCLE’s RoB tool results for each study.

Items of the tool	Ahn YM et al. (2017) [27]	Bachhav SS et al. (2015) [28]	Madlala HP et al. (2015) [29]	Chen S et al. (2017) [31]	Jiang Q et al. (2015) [32]	Luo H et al. (2017) [33]	Pan Y et al. (2018) [34]	Molepo M et al. (2018) [12]	Wang X et al. (2013) [35]	Li Y et al. (2014) [36]	Lee ES et al. (2016) [37]	Wang X et al. (2015) [38]	Gamede M et al. (2018) [39]	Gamede M et al. (2019) [30]	Djeziri FZ et al. (2018) [40]	Nakajima K et al. (2019) [41]	Su S et al. (2018) [42]	Wang S et al. (2018) [43]	An Q et al. (2017) [44]	Matumba MG et al. (2019) [45]	Nyakudya TT et al. (2018) [46]	Nyakudya TT et al. (2018) [47]	Nyakudya TT et al. (2019) [48]
1. Was the allocation sequence adequately generated and applied?	?	?	?	?	+	?	?	?	?	?	?	?	?	—	?	?	?	?	?	?	?	+	+
2. Were the groups similar at baseline or were they adjusted for confounders in the analysis?	+	+	+	+	+	?	+	?	?	+	?	+	+	+	+	?	?	+	?	?	+	+	+
3. Was the allocation to the different groups adequately concealed during?	?	?	?	?	?	?	?	?	?	?	?	?	?	?	?	?	?	?	?	?	?	?	?
4. Were the animals randomly housed during the experiment?	?	+	?	?	?	+	?	?	+	+	+	+	?	?	?	?	?	?	?	?	?	+	+
5. Were the caregivers and/or investigators blinded from knowledge which intervention each animal received during the experiment?	?	?	+	+	?	?	?	?	?	+	?	?	?	?	?	?	?	?	?	?	?	?	?
6. Were animals selected at random for outcome assessment?	?	?	?	?	?	+	?	+	?	?	?	?	?	?	?	?	?	?	?	?	?	?	?
7. Was the outcome assessor blinded?	?	+	?	?	+	+	?	+	?	+	?	+	+	+	+	+	+	+	+	+	?	+	+
8. Were incomplete outcome data adequately addressed?	+	?	?	?	?	+	+	+	?	+	?	+	+	+	+	?	+	+	+	+	?	+	+
9. Are reports of the study free of selective outcome reporting?	+	+	+	+	+	+	+	+	+	+	+	+	+	+	+	+	+	+	+	+	+	+	+
10. Was the study apparently free of other problems that could result in high risk of bias?	?	+	?	?	?	+	+	?	?	+	+	+	+	?	?	?	?	?	?	?	?	?	?

Notes: + (*low risk of bias*);—(*high risk of bias*)*?* (*item not reported, unknown risk of bias*). 1–3 considers selection bias; 4–5 performance bias; 6–7 detection bias; 8 attrition bias; 9 reporting bias; and 10 other biases

**Table 4:** Jadad score results for the clinical trial selected.

Authors (Year)	Randomization	Method of Randomization	Double Blinding	Method of Blinding	Dropouts/Withdrawals	Jadad Score
Luo HQ et al. (2018) [49]	No	No	No	No	Yes	1
